# Supplementary material for: Uncertainty quantification and sensitivity analysis of neuron models with ion concentration dynamics
Source: PLoS One. 2024 May 21;19(5):e0303822. doi: 10.1371/journal.pone.0303822 (PMC11108148; doi:10.1371/journal.pone.0303822)
Supplement: S1 Appendix — (PDF) [file pone.0303822.s001.pdf]

S1 Appendix

MODEL PARAMETERS AND INITIAL CONDITIONS

Table S1. Geometrical parameters

Parameter		Value	Units
Δx	distance between the two layers	6.67×10^{-2}	cm
α	intracellular coupling strength	2	
A_m	membrane area of each cellular compartment	6.16×10^{-6}	cm^2
A_i	intracellular cross-section areas	$\alpha \cdot A_\mathrm{m}$	cm^2
A_e	extracellular cross-section area	6.16×10^{-7}	cm^2
$V_{\mathrm{sn},0}, V_{\mathrm{dn},0}$	initial neuronal volumes	1.437×10^{-9}	cm^3
$V_{\mathrm{se},0}, V_{\mathrm{de},0}$	initial extracellular volumes	7.185×10^{-10}	cm^3
$V_{\mathrm{sg},0}, V_{\mathrm{dg},0}$	initial glial volumes	1.437×10^{-9}	cm^3

Table S2. Diffusion constants, tortuosities, and intraneuronal fractions of mobile ions

Parameter		Value	Units
D_Na	Na^+ diffusion constant	1.33×10^{-8}	$\mathrm{cm}^2 \cdot \mathrm{ms}^{-1}$
D_K	K^+ diffusion constant	1.96×10^{-8}	$\mathrm{cm}^2 \cdot \mathrm{ms}^{-1}$
D_Cl	Cl^- diffusion constant	2.03×10^{-8}	$\mathrm{cm}^2 \cdot \mathrm{ms}^{-1}$
D_Ca	Ca^{2+} diffusion constant	7.1×10^{-9}	$\mathrm{cm}^2 \cdot \mathrm{ms}^{-1}$
λ_i	intracellular tortuosity	3.2	
λ_e	extracellular tortuosity	1.6	
$\gamma_\mathrm{Na}, \gamma_\mathrm{K}, \gamma_\mathrm{Cl}$	intraneuronal fractions of mobile ions	1	
γ_Ca	intraneuronal fraction of mobile ions	0.01	

Table S3. Temperature and physical constants

Parameter		Value	Units
T	absolute temperature	309.14	K
F	Faraday constant	9.6480×10^4	$\mathrm{nC} \cdot \mathrm{nmol}^{-1}$
R	gas constant	8.314×10^3	$\mathrm{pJ} \cdot (\mathrm{nmol} \cdot \mathrm{K})^{-1}$

Table S4. Membrane parameters

Parameter		Value	Units
c_{m}	specific membrane capacitance	3	$\mu\mathrm{F}\cdot\mathrm{cm}^{-2}$
$\overline{g}_{\mathrm{Na,leak,n}}$	Na^{+} neuron leak conductance	0.0246	$\mathrm{mS}\cdot\mathrm{cm}^{-2}$
$\overline{g}_{\mathrm{K,leak,n}}$	K^{+} neuron leak conductance	0.0245	$\mathrm{mS}\cdot\mathrm{cm}^{-2}$
$\overline{g}_{\mathrm{Cl,leak,n}}$	Cl^{-} neuron leak conductance	0.1	$\mathrm{mS}\cdot\mathrm{cm}^{-2}$
$\overline{g}_{\mathrm{Na}}$	maximal conductance of Na^{+} current	30	$\mathrm{mS}\cdot\mathrm{cm}^{-2}$
$\overline{g}_{\mathrm{DR}}$	maximal conductance of K^{+} delayed rectifier current	15	$\mathrm{mS}\cdot\mathrm{cm}^{-2}$
$\overline{g}_{\mathrm{Ca}}$	maximal conductance of Ca^{2+} current	11.8	$\mathrm{mS}\cdot\mathrm{cm}^{-2}$
$\overline{g}_{\mathrm{AHP}}$	maximal conductance of AHP current	0.8	$\mathrm{mS}\cdot\mathrm{cm}^{-2}$
$\overline{g}_{\mathrm{C}}$	maximal conductance of Ca^{2+}-dependent K^{+} current	15	$\mathrm{mS}\cdot\mathrm{cm}^{-2}$
ρ_{n}	Na^{+}/K^{+} neuron pump strength	1.87×10^{-4}	$\mathrm{nmol}\cdot\mathrm{cm}^{-2}\cdot\mathrm{ms}^{-1}$
U_{kcc2}	KCC2 cotransporter strength	1.49×10^{-5}	$\mathrm{nmol}\cdot\mathrm{cm}^{-2}\cdot\mathrm{ms}^{-1}$
U_{nkcc1}	NKCC1 cotransporter strength	2.33×10^{-5}	$\mathrm{nmol}\cdot\mathrm{cm}^{-2}\cdot\mathrm{ms}^{-1}$
$U_{\mathrm{Ca-dec}}$	Ca^{2+} decay rate	0.075	ms^{-1}
$\overline{g}_{\mathrm{Na,leak,g}}$	Na^{+} glial leak conductance	0.1	$\mathrm{mS}\cdot\mathrm{cm}^{-2}$
$\overline{g}_{\mathrm{K-IR}}$	K^{+} glial leak conductance	1.696	$\mathrm{mS}\cdot\mathrm{cm}^{-2}$
$\overline{g}_{\mathrm{Cl,leak,g}}$	Cl^{-} glial leak conductance	0.01	$\mathrm{mS}\cdot\mathrm{cm}^{-2}$
ρ_{g}	Na^{+}/K^{+} glial pump strength	1.12×10^{-4}	$\mathrm{nmol}\cdot\mathrm{cm}^{-2}\cdot\mathrm{ms}^{-1}$
$[\mathrm{Na}^{+}]_{\mathrm{g,threshold}}$	$[Na^{+}]$ threshold for Na^{+}/K^{+} glial pump	1×10^{4}	$\mathrm{nmol}\cdot\mathrm{cm}^{-3}$
$[\mathrm{K}^{+}]_{\mathrm{e,threshold}}$	$[K^{+}]$ threshold for Na^{+}/K^{+} glial pump	1.5×10^{3}	$\mathrm{nmol}\cdot\mathrm{cm}^{-3}$
G_{n}	neuron water permeability	2×10^{-26}	$\mathrm{cm}^{3}\cdot\mu\mathrm{Pa}^{-3}\cdot\mathrm{ms}^{-1}$
G_{g}	glial water permeability	5×10^{-26}	$\mathrm{cm}^{3}\cdot\mu\mathrm{Pa}^{-3}\cdot\mathrm{ms}^{-1}$

Table S5. Basal ion concentrations

Parameter	Value	Units
$[\mathrm{K}^{+}]_{\mathrm{e,b}}$	3.082	mM
$[\mathrm{K}^{+}]_{\mathrm{g,b}}$	99.959	mM
$[\mathrm{Ca}^{2+}]_{\mathrm{n,b}}$	0.01	mM

Note that the system implementation provides basal ion concentrations in $\mathrm{nmol}\cdot\mathrm{cm}^{-3}$.

Table S6. Initial conditions

Variable	Value*	Units
$\phi_{\mathrm{mn},0}$	-66.9	mV
$\phi_{\mathrm{mg},0}$	-83.9	mV
$[\mathrm{Na}^+]_{\mathrm{n},0}$	18.7	mM
$[\mathrm{Na}^+]_{\mathrm{e},0}$	142.3	mM
$[\mathrm{Na}^+]_{\mathrm{g},0}$	14.5	mM
$[\mathrm{K}^+]_{\mathrm{n},0}$	138.1	mM
$[\mathrm{K}^+]_{\mathrm{e},0}$	3.54	mM
$[\mathrm{K}^+]_{\mathrm{g},0}$	101.2	mM
$[\mathrm{Cl}^-]_{\mathrm{n},0}$	7.15	mM
$[\mathrm{Cl}^-]_{\mathrm{e},0}$	131.9	mM
$[\mathrm{Cl}^-]_{\mathrm{g},0}$	5.65	mM
$[\mathrm{Ca}^{2+}]_{\mathrm{n},0}$	0.01	mM
$[\mathrm{Ca}^{2+}]_{\mathrm{e},0}$	1.10	mM
n_0	0.0003	
h_0	0.9993	
s_0	0.0077	
c_0	0.0057	
q_0	0.0117	
z_0	1.0	

Note that ϕ_{mn} and ϕ_{mg} are not independent state variables, but calculated from the ion concentrations at each time step. Their "initial conditions" are used to calculate the amount of immboile ions in the system. Moreover, the system implementation provides initial conditions for ion dynamics expressed in nmol.
* Values with more decimals included were read to/from file and used in the simulations.
