# Supplementary material for: Uncertainty quantification and sensitivity analysis of neuron models with ion concentration dynamics
Source: PLoS One. 2024 May 21;19(5):e0303822. doi: 10.1371/journal.pone.0303822 (PMC11108148; doi:10.1371/journal.pone.0303822)
Supplement: Attachment — Submitted filename: response_to_reviewers.pdf [file pone.0303822.s002.pdf]

Dear Editor,

We hereby resubmit our manuscript "Uncertainty quantification and sensitivity analysis of neuron models with ion concentration dynamics" by Letizia Signorelli, Andrea Manzoni, and Marte J. Sætra for publication in PLOS ONE.

We thank the reviewers for their feedback and constructive comments. Our detailed response follows below. The editor's and reviewers' comments are included in blue, while our responses follow in black text. Also attached is a version of the manuscript indicating the changes made from the previous version.

Editor: First, reviewers have raised concerns on model calibration. Therefore, the choice of parameter values requires better justification.

Comment: Reviewer 1 had a question about the categorization of parameters into the "non-dynamic" and "dynamic" parameter groups. To address this question, we have added the necessary explanations to the manuscript, as detailed in our response to the reviewer.

Editor: Second, reviewers found multiple typos and convoluted spots. Therefore, the manuscript is required to be corrected and carefully proofread by a proficient English speaker.

Comment: A careful check on the whole manuscript has been carried out in this respect.

Editor: Additionally, the manuscript would improve if the discussion was written for a more general audience.

Comment: Reviewer 2 suggested that we should add more discussions/explanations to the manuscript on how the different challenges were solved to make it easier for others to learn from our case study. We fully agree that this would improve the paper as we want the manuscript to offer guidelines for others to follow. To address this suggestion we have added several new paragraphs throughout the paper as detailed in our response to the reviewer.

Reviewer #1: This paper carried out uncertainty quantification analysis for a six-compartment neuron model incorporating ion concentration dynamics from a comprehensive viewpoint. The topic is interesting and important, and the English language is clear. I think the paper can be accepted after a minor revision.

Comment: We thank the reviewer for this prompt and positive evaluation of our manuscript.

Reviewer #1: I am wondering how the dynamical parameters and the non-dynamical parameters are taken in Table 1 and Table 2. The authors should add necessary demonstration or citation.

Comment: Thanks for the opportunity to clarify this. The initial categorization of the parameters, which we subsequently confirmed with our factor fixing sensitivity analysis,

were made through an educated guess based on the edNEG model's biophysics and a preliminary sensitivity analysis of the analogous edPR model. To clarify this first step, we have rephrased the first sentence of the Factor fixing Procedure description:

Line 243: "Based on the edNEG model's biophysics, and informed by a preliminary and previously done sensitivity analysis of the analogous edPR model [40], we made an educated guess to categorize the parameters of interest into two distinct groups."

Furthermore, to underscore the aim of this categorization/factor fixing, we added the following sentence at the end of the Factor fixing Procedure description:

Line 253: "The aim of this factor fixing procedure was to ensure that the "dynamic" parameters did not affect the resting state, allowing us to subsequently concentrate on examining how uncertainty in these parameters uniquely influences firing dynamics."

Reviewer #1: The authors should reexamine their whole context to eliminate the possibles errors. For example, in the abstract, the sentence "To mitigate computational cost, we employ surrogate modeling techniques, optimized using efficient numerical integration techniques." contains grammatical error due to "optimized using efficient numerical integration techniques". For another example, the sentence "Our sensitivity analysis not only sheds some light on the critical parameters influencing our model's outputs, their interdependencies, and which ones demand precise estimation to mitigate uncertainty in the results" seems strange due to " and which.....", please see Line 638-640.

Comment: Thanks for the remark. A careful check on the whole manuscript has been carried out in this respect.

Reviewer #2: This paper investigates the effect of uncertain parameters on an electrodiffusive neuron-extracellular-glia (edNEG) neuron model through the use of uncertainty quantification (UQ) and sensitivity analysis (SA). The authors focus on the effect of uncertainties in a selected set of dynamic model parameters and examined the effects on the model under two different conditions, physiological and pathological. This paper discusses several common challenges for uncertainty quantification of neuron models with ion concentration dynamics and is a good showcase for how to approach these challenges within the field of neuroscience.

The main strength of the paper is the case study and discussion of the encountered challenges. This serves as a showcase for how common challenges related to UQ and SA of neuron models may be addressed, contributing to wider adoption of uncertainty quantification within neuroscience.

There are a few minor issues that preferably could be addressed to improve the article, but no major issues that fundamentally affect the work and conclusions.

Comment: We thank the reviewer for this prompt and positive evaluation of our manuscript.

Reviewer #2: The main issue that would strengthen the paper if it was improved is to generalize the discussions related to how the different challenges were solved, making it easier for others to apply them, as the authors mention they want the article to offer guidelines for others to follow.

One example is the section on "Numerical implementation and validation" of the edNEG model (line 165), which details how the edNEG model was optimized in order to perform UQ and SA. The specific optimizations needed are of course highly dependent on the specific model and could be the topic of multiple focused papers, however it would be useful to have more information why these specific changes were made and the thought process behind them, as that will help guide others. It would also be useful if the text could mention some general considerations that others can make. For example, why was the choice of a timestep of 10ms selected? What other timesteps did the authors consider to use and why were they discarded?

Comment: Thanks for the suggestion. We agree that such discussions will improve the manuscript. We have expanded the section on Numerical implementation and validation with the following descriptions:

Line 168: "...we successfully improved convergence by rescaling units and implementing an analytical Jacobian. The first strategy aimed at confining the range of orders of magnitude for the state variables. The second strategy sped up computations and improved result accuracy. Specifically, the analytical Jacobian prevented approximation errors resulting from SciPy's default finite difference approximation. These changes made implicit solvers work effectively, and resulted in simulations that were up to fifteen times faster than the original ones, depending on the choice of integration method and maximum time step. We subsequently conducted a convergence analysis of the number of action potentials and time of the last action potential for phi_msn, the extracellular potassium concentration $[K^{+}]$_se, and the extracellular volume V_se for solve_ivp's different solvers. As a result of this analysis, for UQ and GSA, we opted for the implicit solver Radau with a maximum time-step length of 10 ms. Note that the solve_ivp function utilizes adaptive time stepping, and delta t_max indicates the maximum allowed time step. Our choice balanced computational efficiency with the necessity to effectively capture dynamics within a simulation time of T = 6 s. Our selection of the Radau solver was primarily due to its implicit nature, which allows accurate results to be obtained with larger time-step lengths. Additionally, it proved to be more accurate at large time-step lengths for our particular model compared to other SciPy implicit solvers."

Reviewer #2: The same applies elsewhere, it would for example be useful to know why the selected quantities of interest were chosen for examination in the physiological and pathological cases (line 298-305). If the reader is provided with insight as to why these quantities of interest were the most relevant for this case study, it may be easier for them to make similar considerations for their own use case.

Comment: Agree. We added the following paragraph to the QoIs (for the dynamical state) section:

Line 336: "The choice of QoIs will naturally depend on the type of study being conducted. Since the goal of this study is to demonstrate how to perform UQ and GSA on neuron models with ion concentration dynamics, we choose to focus on general spiking features capturing the neuronal firing pattern when studying the membrane potential. To represent slow dynamics, we select $[K^+]$_se because of its crucial role in depolarization blocks [19]. For those interested in exploring other spiking features such as the width or height of the APs, or slow dynamics variables like other ion concentrations or volumes, they can readily apply the same methodology that we have used."

Reviewer #2: There might also be other places where broader perspectives could enhance the article, perhaps for the Factor fixing section (line 231)?

Comment: Yes. We have expanded the Factor fixing QoI section with the following explanation:

Line 274: "We selected these QoIs because temporally constant membrane potentials and ion concentrations characterize the system's resting state. If a parameter is altered, the membrane potentials and ion concentrations may deviate from an initial resting state until the system stabilizes in a new resting state."

In addition, we have clarified how the parameters were categorized into the "non-dynamic" and "dynamic" parameters group as described in one of our comments to Reviewer 1.

Reviewer #2: The first sentence of the abstract makes me interpret the paper to focus on inventing new computational or algorithmic approaches for more efficient UQ and SA. It would be beneficial with a rephrasing that shows the main strength of the paper, namely as a case study for how common challenges with UQ and SA in neuroscience can be approached and solved.

Comment: We agree, thanks for the remark. The sentence has now been rephrased:

"This paper provides a comprehensive and computationally efficient case study for uncertainty quantification (UQ) and global sensitivity analysis (GSA) in a neuron model incorporating ion concentration dynamics. We address how challenges with UQ and GSA in this context can be approached and solved, including challenges related to…"

Reviewer #2: 53-54: "To fully exploit the potential of surrogate modeling, it is essential to adopt efficient numerical integration techniques". Would this not be even more essential if other methods than surrogate modeling were used, for example quasi-Monte Carlo methods?

Comment: Thanks for the remark. We have restructured the paragraph since it could be misunderstood. We now talk about the model complexity and numerical challenges first,

before moving on to talk about the cost of running numerous input-output evaluations (line 40).

Reviewer #2: 82-83: “Additionally, evaluating sensitivity indices for a time-dependent output (i.e., one for each time point) is computationally demanding,”. I am just curious, is this computationally demanding when compared to the computational cost of running the model evaluations?

Comment: With that sentence, we wanted to specify that evaluating sensitivity indices for a time-dependent output (i.e., one for each time point) is computationally demanding compared to a scalar output, such as evaluating spiking features.

Reviewer #2: 85-86: “we introduce a comprehensive and computationally efficient approach for conducting UQ and GSA on neuron models with ion concentration dynamic”. This sentence gives the same impression as in the abstract (1.). Perhaps rephrasing it would make the content of the paper more clear?

Comment: Agree. We have rephrased the sentence:

Line 83: “In this paper, we present a comprehensive and computationally efficient case study for UQ and GSA on a neuron model with ion concentration dynamics.”

Reviewer #2: 99 - 100: “This efficiency demonstrates the feasibility of conducting sensitivity analysis on complex neuroscience models”. It would be beneficial to know the runtime for UQ and SA of the model with and without the performance improvements, since most readers likely do not know the performance of the unoptimized model and subsequently if the (rather impressive) speedup is necessary.

Comment: We agree, thanks for the remark. We have added a new sentence to clarify the analysis runtimes:

Line 351: “The UQ and GSA runtimes varied between 30 minutes for physiological conditions with scalar output and 120 minutes for pathological conditions with time-dependent output. Timings were conducted on an Acer SPIN 5 SP513-52N with an Intel Core i5-8250U CPU running at 1.60GHz and 4 cores, using Python 3.8.”

We did not run the analysis using the unoptimized model since we currently don’t have a setup for this, but we assume that it would take about 15 times longer.

Reviewer #2: 178-179: The code is readable and freely available with instructions for how to run it, which is very good! My one suggestion would be to also archive the code on zenodo (https://zenodo.org/). That would give the code a DOI to cite and track it with and provide redundancy in case github repositories get deleted.

Comment: Good idea. We have made a new release of the code and archived it on Zenodo as you suggested (https://doi.org/10.5281/zenodo.10775265). We chose to keep the references to Github in our manuscript, but have added an additional citation to the Zenodo repo.

Reviewer #2: 248: Was there a reason an uncertainty of 15% was used in the factor fixing analysis, while for examining the dynamic parameters {0, 1, 5, 10} was used?

Comment: The reason behind the choice of uncertainty being 15% in the factor fixing analysis lies in the different simulations conducted. While the factor fixing analysis focused on the model in its resting state, the examination of dynamic parameters involved subjecting the system to a stimulus current, inducing spiking dynamics. The choice of 15% uncertainty in the former case aimed to better identify changes in the resting state, while during the spiking dynamics a higher level of uncertainty introduced excessive variability. Furthermore, our objective when examining the dynamic parameters was to conduct a study encompassing low (1%), medium (5%), and high (10%) levels of uncertainty.

Reviewer #2: 258: "these variables were carefully selected": It would be nice with a reference to where in the paper this ends up being expanded upon (see comment on main issue).

Comment: Agree. We removed the statement saying "these variables were carefully selected" and added the following explanation instead:

Line 274: "We selected these QoIs because temporally constant membrane potentials and ion concentrations characterize the system's resting state. If a parameter is altered, the membrane potentials and ion concentrations may deviate from an initial resting state until the system stabilizes in a new resting state."

Reviewer #2: 261-266: "Implementation details". To my understanding, this calculates the Sobol indices using quasi-Monte Carlo methods? Why are not surrogate models used, as that is mentioned earlier to be imperative for computational efficiency? It would also make the text more clear if the authors mention that the goal is to calculate the Sobol indices.

Comment: The reviewer is correct: in the factor-fixing analysis, surrogate models are not used. This is because it is a preliminary analysis conducted with the model in resting conditions and with scalar outputs, hence not requiring a surrogate. We added the following sentence to clarify:

Line 284: "Since the factor-fixing analysis was a preliminary analysis conducted with the model in resting conditions and with scalar outputs, it did not require the use of surrogate models."

The Sobol' indices are mentioned in the Procedure description- (line 253), Quantities of interest- (line 274), and Implementation details paragraphs (line 283).

Reviewer #2: 319-320: “Outputs were generated by drawing a Monte Carlo sample of size 104 from the parameter distribution”. To my understanding, this sentence refers to how many samples were used for finding the 5th and 95th percentiles? (the nr_pc_mc_samplest argument?). If so, it might be more clear to say 5th and 95th percentiles instead of outputs, and I would put it after the next sentence “To enhance computational efficiency,” as that is the more important information in this context.

Comment: The reviewer is right. We decided to remove the sentence altogether, as it is the default setting and not necessary to know in our context.

Reviewer #2: 323: “Rosenblatt transformation for dependent parameter”, the Rosenblatt transformation is not necessary unless there are dependencies in the input parameters, which seems to not be the case in the manuscript?

Comment: The reviewer is right. We decided to remove the Rosenblatt transformation and update the figures since it is not necessary.

Reviewer #2: 350: It is a bit unclear to me how the total order Sobol indices for each group was found in the factor fixing analysis, is it the sum of the Sobol indices for all uncertain parameters in each group?

Comment: To compute the Sobol’ indices for each group, we relied on the Salib library (https://salib.readthedocs.io/en/latest/#), where this feature is implemented. The difference with respect to the usual computation of Sobol' indices lies in how the matrices are cross-sampled when generating the Saltelli low-discrepancy sequence. Instead of having two matrices NxD with half of the samples (N samples and D number of input parameters) and D matrices of input realizations NxD, only Dg matrices of input realizations are generated (where Dg is the number of groups). This results in a total of (Dg + 2) * N model evaluations instead of (D + 2) * N. Sobol’ indices are then computed directly with respect to each group of parameters.

Reviewer #2: Fig 3, Fig 5, Fig 6, and Fig 8 could all benefit from having subtitles added to the subplots. The plots are readable, but that would make it easier and faster to interpret them.

Comment: Fixed.
